# A prospective investigation of the effects of soccer heading on cognitive and sensorimotor performances in semi-professional female players

**DOI:** 10.3389/fnhum.2024.1345868

**Published:** 2024-02-09

**Authors:** Jan Kern, Philipp Gulde, Joachim Hermsdörfer

**Affiliations:** Chair of Human Movement Science, Department Health and Sport Sciences, TUM School of Medicine and Health, Technical University of Munich, Munich, Germany

**Keywords:** soccer heading, (repetitive) head impacts, cognition, sensorimotor performance, females

## Abstract

**Introduction:**

Repetitive head impacts (RHI) from routine soccer (football) heading have been suggested to contribute to the long-term development of neurodegenerative disorders. However, scientific evidence concerning the actual risk of these RHI on brain health remains inconclusive. Moreover, female athletes—despite a presumably increased vulnerability toward the effects of RHI—are largely underrepresented in previous approaches. Therefore, our aim was to prospectively investigate the effects of heading on cognitive and sensorimotor performances, health perception, and concussion symptoms in semi-professional female soccer players.

**Methods:**

An extensive test battery was used to assess cognitive and sensorimotor performances as well as health status (SF-36) and concussion symptoms (SCAT3) of a total of 27 female soccer players (22.2 ± 4.2 years) and 15 control subjects (23.2 ± 3.0 years) before and after one-and-a-half years. Throughout this period, soccer players’ heading exposure was determined using video analysis.

**Results:**

Subgroup comparisons (control [*n* = 12], low exposure [*n* = 7], high exposure [*n* = 8]) showed no time-dependent differences in SF-36 or SCAT3 scores. Similarly, across most behavioral tests, soccer players’ performances evolved equally or more favorably as compared to the control subjects. However, there were significant effects pointing toward slightly negative consequences of heading on aspects of fine motor control (*p* = 0.001), which were confirmed by correlation and multiple regression analyses. The latter, further, yielded indications for a relationship between heading exposure and negative alterations in postural control (*p* = 0.002).

**Discussion:**

Our findings do not provide evidence for negative effects of soccer heading on female players’ health perception, concussion symptoms, and cognitive performances over the course of one-and-a-half years. However, we found subtle negative alterations in fine motor and postural control that could be attributed to heading exposure. Other factors, like the number of previous head injuries, were not linked to the observed changes. Given the reduction of our initial sample size due to player fluctuation, the results need to be interpreted with caution and validated in larger-scale studies. These should not only focus on cognitive outcomes but also consider sensorimotor changes as a result of RHI from soccer heading.

## Introduction

There exists growing concern about the potentially adverse long-term consequences of sport-related head impacts, especially with respect to the proposed association between recurrent concussions and the late development of neurodegenerative disorders, such as dementia or chronic traumatic encephalopathy (CTE) (Cunningham et al., 2020). Much of this attention stems from the observation of these pathologies in (retired) contact sport athletes like American football players (McKee et al., 2013) and boxers (Mendez, 1995). However, recent studies have reported similar findings, along with an increased mortality from neurodegenerative disease (Mackay et al., 2019), among former soccer (football) players (Grinberg et al., 2016; Ling et al., 2017). While rates of concussion in the game of soccer are considerably lower when compared to other contact sports (Prien et al., 2018), soccer players actively expose themselves to repetitive head impacts (RHI) by purposefully heading the ball. Although these RHI, by definition, do not lead to acute clinical signs and symptoms and, thus, are commonly referred to as “subconcussive” head impacts (Bailes et al., 2013), it is suspected that the subtle effects of numerous soccer headers might accumulate over several years of play and, thus, contribute to the development of long-term neurological deficits (Gavett et al., 2011; Koerte et al., 2015).

Since the earliest reports of chronic traumatic brain injury (Sortland and Tysvaer, 1989) and cognitive impairment (Tysvaer and Løchen, 1991) in professional soccer players, an increasing amount of research has focused on uncovering the potential effects of soccer heading on brain structure and function. While laboratory studies that aim toward the investigation of immediate, yet transient, perturbations of brain function following a controlled bout of soccer heading are imperative to elucidate the direct effects of heading following a training session, these cross-sectional approaches neither represent heading characteristics nor actual heading frequency during competitive play and, thus, do not allow for a proper investigation of potential long-term consequences of soccer heading (Tarnutzer, 2018). To assess these presumably cumulative or persistent effects, studies usually focus on the examination of retired soccer players (Prien et al., 2020; Haase et al., 2023) or employ observational designs, in which athletes’ functional, structural, or metabolic brain alterations are prospectively evaluated over the course of several matches up to single seasons (Lipton et al., 2013; Amitay et al., 2020). Within this context, findings on potentially detrimental cumulative or long-term consequences of soccer heading appear to be highly inconsistent. While there are reports on neuroimaging abnormalities (Koerte et al., 2015) and memory deficits (Koerte et al., 2016) in former soccer players, other studies found no unequivocal evidence for cognitive impairment or dementia among retired athletes (Prien et al., 2020; Haase et al., 2023). Likewise, a dose-response-relationship between cumulative heading over a season and the degree of cognitive dysfunction—as particularly manifested in memory deficits—in active players has been reported by some authors (Matser et al., 2001; Lipton et al., 2013), whereas others found no such associations (Rutherford et al., 2009; Amitay et al., 2020).

While these ambiguous results do not allow for definitive statements on whether soccer heading is associated with lasting adverse health outcomes, two systematic reviews argue that this lack of conclusive empirical evidence is mainly due to several methodological shortcomings of previous studies (Rodrigues et al., 2016; Tarnutzer et al., 2017). Among others, these include failure to control for subjects’ potential history of previously sustained concussions, selection bias owing to retrospective research designs, and, above all, low-quality assessment of heading frequency by means of self-report questionnaires, which does not allow to establish an objective link between players’ individual heading exposure and potential functional and / or structural deficits (Rodrigues et al., 2016; Tarnutzer et al., 2017). Furthermore, the majority of previous research on the potentially adverse effects of soccer heading has focused on male athletes (Tarnutzer et al., 2017). As the number of women actively participating in soccer continues to increase (Rodrigues et al., 2016), this can be considered an important gap in the literature (Prien et al., 2020). Moreover, the findings of several studies indicate that women may be particularly vulnerable to repetitive subconcussive head impacts. Bretzin et al. (2017) and Caccese et al. (2018) could show that, during a controlled heading intervention, female soccer players experience significantly greater head accelerations than males. Further, Rubin et al. (2018) demonstrated that, with similar exposure to heading, females show more extensive microstructural white matter alterations when compared to males. Consequently, there is well-founded concern that repetitive headers may have lasting effects on female soccer players’ brain health.

Against this assumption, the few prospective studies that explicitly focused on female players found no evidence for long-term changes in cognitive, sensorimotor or autonomic function as a result of cumulative soccer heading (Kaminski et al., 2007, 2008; Wahlquist et al., 2019; Bonn et al., 2021). However, as these studies were conducted in either youth (Bonn et al., 2021), high school (Kaminski et al., 2007, 2008; Wahlquist et al., 2019), or college (Kaminski et al., 2007) settings, it could be argued that overall exposure to soccer play and purposeful heading has been relatively low, not reaching levels that may be necessary to induce measurable or clinically relevant alterations (Tarnutzer et al., 2017). Indeed, in their samples of female youth and high school players, Kaminski et al. (2007, 2008), and Bonn et al. (2021) reported an average number of approximately only one header per player per match. Therefore, it may be assumed that female players performing on a higher level (e.g., semi-professional) are exposed to a greater number of headers and, thus, face an increased risk toward functional brain changes due to repetitive heading as a result of their more extensive soccer exposure (Tarnutzer et al., 2017).

Considering the presumed vulnerability of women toward RHI and the presented lack of prospective approaches to examine the potentially adverse effects of soccer heading, especially in high-level female athletes, the aim of this study was to investigate a potential relationship between female players’ heading exposure and changes in cognitive and sensorimotor function. To that aim, we used an extensive test battery to assess and compare health status, concussion symptoms, and, above all, cognitive and sensorimotor performances of female soccer players and female athletes participating in non-contact sports before and after one-and-a-half soccer seasons. Throughout this period, we collected individual header data of the soccer players by means of analyzing video footage of competitive matches. With this prospective approach, we expected to allow for an accurate determination of individual heading exposure and enable an appropriate investigation of functional brain changes induced by soccer heading.

## Materials and methods

### Subjects

A total of 27 female soccer players (mean age: 22.2 ± 4.2 years) and 15 female athletes (mean age: 23.2 ± 3.0 years), participating in the non-contact sports of volleyball (*n* = 11), gymnastics (*n* = 2), and triathlon (*n* = 2), took part in the study. Soccer players were part of a semi-professional female team competing in the German 3rd division and encompassed all out-field positions (defenders [*n* = 10], midfielders [*n* = 8], forwards [*n* = 7]) and two goalkeepers. Control subjects were recruited from athletic teams within the greater Munich area and were required to coarsely match the soccer players with respect to age, years of training, and training hours per week (Table 1). To be included in the study, all subjects had to be free of neurological, psychiatric, and neurodevelopmental conditions (e.g., attention deficit and hyperactivity disorder [ADHD] or learning disorders) as assessed using self-reports. Further, a potential history and recurring symptoms of migraine were assessed using the migraine disability assessment (MIDAS) (Stewart et al., 2001). All participants scored < 2 points on the MIDAS, indicating little to no impairment due to migraine or its related symptoms (Stewart et al., 2001). Subjects with a self-reported history of sustained head injuries were included, as long as the last head injury occurred > 12 months prior to the study and participants reported no persisting symptoms. Of the 27 soccer and 15 non-contact sport athletes, 10 (35.7 %) and 5 (33.3 %) reported suffering at least one head injury prior to the study, respectively. Of the soccer players with a history of sustained head injuries, 7 (70 %) reported a single head injury, while the remaining ones reported 2, 3, and 4 head injuries, respectively (i.e., 10 % each). The control athletes with a history of sustained head injuries each reported to have suffered a single head injury. Sample characteristics, including athletic experience and history of sustained head injuries, are presented in Table 1. All subjects provided written informed consent to participate in the study, which was approved by the ethical committee of the School of Medicine of the Technical University of Munich and conducted in accordance with the declaration of Helsinki.

**TABLE 1:** Sample characteristics.

	Soccer players	Controls	*p*
Subjects [n]	27	15	–
Age [y]	22.2 (± 4.2) [18–31]	23.2 (± 3.0) [20–29]	0.404
Height [cm]	168.1 (± 4.0) [163–177]	169.0 (± 5.6) [159–176]	0.568
Mass [kg]	62.5 (± 5.0) [49–72]	63.8 (± 6.0) [53–75]	0.451
BMI [kg/m^2^]	22.1 (± 1.8) [18.2–25.8]	22.3 (± 1.1) [21.0–24.5]	0.722
Years of training [y]	15.6 (± 5.1) [7–27]	13.3 (± 4.5) [9–27]	0.155
Training/week [h]	4.8 (± 1.4) [2–8]	4.8 (± 1.6) [3–9]	0.969
Head injuries [n]	0.6 (± 1.0) [0–4]	0.3 (± 0.5) [0–1]	0.357
**Educational level^a^**			
No degree	0 (n.a.)	0 (n.a.)	0.862
General secondary school	0 (n.a.)	0 (n.a.)	
Intermediate secondary school	4 (14.8 %)	1 (7 %)	
High school	12 (44.4 %)	7 (47 %)	
Vocational school	4 (14.8 %)	2 (13 %)	
University	7 (25.9 %)	5 (33 %)	

^a^Highest degree according to the German educational system. Values depicted as mean (± SD) [range], except for educational level, which is displayed as absolute numbers (%), *p*-values as revealed by independent *t*-tests or X^2^ test (educational level).

### Procedure

The study was conducted over a period of one-and-a-half years (December 2017–July 2019), with baseline testing taking place before the winter break of the soccer season. In a comprehensive assessment, subjects of both groups, first, provided general information on demographic (age, educational level), anthropometric (body height, body mass), injury-/ disease-specific (number of previous [head] injuries, neurological, psychiatric, and neurodevelopmental conditions, medication) and sport-related characteristics (training hours, years of sports participation, previous participation in contact sport, playing position) by means of self-reports. Further, subjects’ self-perceived health status and concussion symptoms were assessed using the Short Form Health Survey (SF-36) (Ware, 1996) and the Sport Concussion Assessment Tool 3 (SCAT3) (Guskiewicz et al., 2013), respectively. At the beginning of the study, the SCAT3 was the most recent version of the SCAT questionnaires. While, in the meantime, updated versions have been released—such as the SCAT6 in 2023 (Echemendia et al., 2023)—the assessment of self-perceived concussion symptoms does not differ between the SCAT3 and the SCAT6. Finally, athletes’ cognitive and sensorimotor performances were evaluated using an extensive test battery consisting of both computerized and paper-and-pencil tests. As there exists no consensus which cognitive and sensorimotor domains may be particularly affected by RHI, our general aim was to capture subjects’ performances across a broad range of functional domains and, moreover, use tests with a high sensitivity toward identifying subtle cognitive and sensorimotor deficits as well as specificity in detecting impairments typically associated with (sub-)concussive head impacts. More specifically, our selection of cognitive tests was based on previous research (e.g., Koerte et al., 2015; Ashton et al., 2021) that examined the acute or cumulative effects of soccer heading on athletes’ cognitive functioning. Given that sensorimotor changes (with the exception of alterations in postural control) have only rarely been examined as a potential result of repetitive soccer heading, but are commonly to be observed following concussion (De Beaumont et al., 2011), we decided to also focus on the domains of fine motor control, manual dexterity, grip force and postural control as these have been reported to be affected by subconcussive head impacts other than soccer headers or concussions (Brokaw et al., 2018; Servatius et al., 2018; Caccese et al., 2021).

To reliably quantify the soccer players’ individual number of headers throughout the study period, video footage of competitive soccer matches was captured by means of two high-definition video cameras (Sony HDR-CX653, Sony Corp., Tokyo, Japan) recording with 50 fps at 1080p resolution. Each camera was mounted on a tripod and located on opposing sides of the pitch near the midpoint, with each of them capturing on side of the pitch. Video footage was reviewed and analyzed by a trained researcher using Kinovea 0.8.27^1^ in order to identify the headers performed by each soccer player. Moreover, the researcher was instructed to have high sensitivity in identifying unintentional head impacts (e.g., head-to-head, head-to-ground, head-to-goalpost) to be able to account for the effects of potential concussions. Besides the mere quantification of header events, we also classified headers according to the distance, which the ball travelled before the header was performed. In line with previous research (Sandmo et al., 2020; Reeschke et al., 2023), this was done to obtain an estimate of impact intensity, since not only the pure amount, but also the intensity of headers might be considered when examining their potentially adverse effects. Within this context, headers following short distance passes (< 20 m) or bounces were considered low-intensity headers, whereas headers that were performed from passes > 20 m (e.g., goal-kicks, corners, etc.) were considered high-intensity headers. Overall, we observed a ratio of 58 % low-intensity vs. 42 % high-intensity headers. Interestingly, this ratio was also retained on an individual level, along with a rather low interindividual variability (SD = 6 % for low-intensity and high-intensity headers) between players. Due to this consistent heading behavior between players in terms of intensity, we decided to only consider differences in the number of performed headers in our analysis. Of the 39 competitive matches that took place throughout the observational period, 37 were analyzed. For the remaining two matches, no video data could be collected due to camera / battery malfunction. Since the soccer team’s training sessions did not encompass any specific heading drills, as reported by the coaching staff, quantification of players’ individual heading exposure was restricted to competitive matches, assuming that the overall number of headers during training was low and the relative heading frequency of different players was similar to that in competition. To ensure a single rater was appropriate to analyze video data, a random subset of five matches was selected to be reviewed by a second independent researcher. The interrater reliability in identifying players’ individual numbers of headers was assessed by computing the intraclass correlation coefficient (ICC).

After the end of the first soccer season (13 matches), the group of soccer players took part in a second assessment (re-test) of cognitive and sensorimotor performances, which comprised the same behavioral tests as the initial measurement session. Additionally, two tests for the assessment of executive functioning were added at the end of the original test battery in order to examine a broader range of potentially relevant cognitive and sensorimotor skills. Due to organizational constraints this re-test was restricted to the group of soccer players. Following another season (26 matches), subjects of both groups underwent a final examination (post-test) of cognitive and sensorimotor performances. Before each assessment session, athletes in both groups were asked about physical and mental conditions that may affect their cognitive or sensorimotor test performances—e.g., lower-extremity ligament injuries during the assessment of postural control. However, none of the subjects reported on any conditions potentially affecting their performances. Supplementary Figure 1 provides an overview of the data collection process and the number of players (including drop-outs and drop-ins) that took part in the assessments at each time-point. Cognitive and sensorimotor tests procedures are described in the following and summarized in Tables 2, 3, respectively.

**TABLE 2:** Cognitive tests and corresponding score parameters.

Test	Subtests	Domain	Score parameters	Type
TMT	B	Visual capacity, task-switching ability	Duration [s]	Paper-and-pencil
WDST	fw bw	Short-term memory, working memory	Sum of correct strands from both subtests [n]	n.a.
2Back-Task	–	Working memory	RT [ms] Accuracy [%]	Computerized
Simon Task*	–	Response inhibition, complex attention, processing speed	Simon Effect [ms] Accuracy [%]	Computerized
Stroop Test*	–	Response inhibition, complex attention, processing speed	Stroop Effect [ms] Accuracy [%]	Computerized

TMT, Trail Making Test; WDST, Wechsler Digit Span Test; fw, forward; bw, backward; RT, reaction time; n.a., not applicable;

*Tasks were added to the test battery for the re-test.

**TABLE 3:** Sensorimotor tests and corresponding score parameters.

Test	Subtests	Domain	Score parameters	Type
Writing	Sentence (S) Circle (C)	Fine motor control	SFreq [Hz], SPress [N] CFreq [Hz], CPress [N]	Paper-and-pencil
Grip force	Tracking Force change	Grip force control	Error [RMS] Frequency [Hz]	Computerized
9HPT	–	Manual dexterity	Duration [s]	n.a.
Balance	Stable, v− Instable, v+ DT	Postural control	CoPV [cm/s] CoPDist [cm]	n.a.

S, sentence; C, circle; Freq, frequency; Press, pressure; Track, tracking; RMS, root mean square; FC, force change; 9HPT, 9-Hole-Peg Test; v−, eyes closed; v+, eyes open; DT, dual-task; CoP, center of pressure; V, velocity; Dist, distance; n.a., not applicable.

### Cognitive testing

Visuospatial capacity and task-switching ability were assessed by means of the Trail Making Test (TMT) part B (Tombaugh, 2004), in which subjects had to connect 13 numbers and letters in alternating and ascending order (1, A, 2, B, 3, C, …) as quickly as possible. The TMT was performed with a standardized task sheet fixed on a Wacom Intuos IV digitizing tablet (Wacom Co., Saitama, Japan), which captured trial duration.

The forward and backward parts of the Wechsler Digit Span Test (WDST; Psychological Corporation, San Antonio, USA) were used to assess short-term and working memory. Participants were asked to repeat a series of number strands that increased in length, first in forward and then in reverse order. The sum of correctly reproduced strands from both conditions served as outcome parameter.

Response inhibition, complex attention, and processing speed were examined by means of the Simon Task (Simon and Rudell, 1967). A green or red square was displayed on either the left or right side of a computer screen using Presentation software (Neurobehavioral Systems).^2^ Subjects were instructed to press the left “Alt” key each time a green square appeared and the right “Alt” key each time a red square appeared, irrespective of the location of the square. For congruent (C) trials, the response key was on the same side as the stimulus, whereas for incongruent (IC) trials, the response key was on the opposite side of the stimulus. Athletes had to respond as quickly and accurately as possible to a total of 80 stimuli (40 C and 40 IC in randomized order). The difference in mean reaction times between C and IC trials (Simon Effect) as well as the proportion of correct responses across trials (Accuracy) were used as outcome variables.

A computerized version of the Stroop Test (Stroop, 1935) was administered to assess response inhibition, complex attention, and processing speed. Two types of color stimuli were presented on a computer screen and the subjects’ task was to respond to the color (blue, green, red, or yellow) in which each stimulus was printed by pressing the corresponding color-coded key on a computer keyboard. Neutral stimuli (N) were a series of “x” letters (“xxxxx”), whereas incongruent stimuli (IC) consisted of the words blue, green, red, or yellow, each printed in a different color compared to their semantic meaning. Participants were instructed to respond as quickly and accurately as possible to a total of 48 N and 48 IC stimuli that were presented in randomized order. The difference in mean reaction times between N and IC trials (Stroop Effect) as well as the proportion of correct responses across trials (Accuracy) were used as outcome parameters.

### Sensorimotor testing

Assessment of fine motor control was carried out using a Writing Analysis on a Wacom Intuos IV digitizing tablet and a pressure-sensitive stylus. The first subtask comprised three trials, across which subjects had to write the German sentence “Die Wellen schlagen hoch” (“The waves are surging high”) on a blank sheet of paper fixed upon the tablet. In the second subtask, participants were asked to produce superimposed circles as fast as possible for three seconds across three trials. Although the analysis of handwriting, to date, has not been commonly used to assess sensorimotor deficits following RHI or concussion, writing deficits have been previously reported after brain injury (Faddy et al., 2008; Titchener et al., 2018). Moreover, we opted for the inclusion of these two subtasks, as handwriting generally constitutes a fine motor skill with a high degree of automatization and both subtasks have been previously identified as highly sensitive measures for the assessment of sensorimotor deficits (Hermsdörfer et al., 2011). For both subtasks, the average number of up and down strokes per second (Freq) and the mean pressure (Press) exerted onto the tablet by the tip of the stylus were used as performance parameters. Within this context, studies showed that both parameters are very sensitive to detect subtle changes in the fine motor control of handwriting (Garre-Olmo et al., 2017). Moreover, negative alterations in pen pressure have been reported in individuals suffering from parkinsonism (Saini and Kaur, 2019)—a clinical syndrome commonly linked to RHI in retired contact sport athletes, including soccer players (Mackay et al., 2019).

Grip Force Control was assessed across two subtasks using a custom-built grip force sensor (71 mm × 57 mm × 22 mm; 180 g). Participants were asked to grasp the device with the tips of the thumb and three fingers of their dominant hand (index-, middle- and ring finger) in opposition. In both subtasks, the grip force exerted on the manipulandum was represented as a vertical bar on a computer screen in front of the subjects. In a visuomotor tracking subtask, athletes had to align the top of the bar to a randomly vertically moving horizontal line by adjusting their grip force accordingly. Five 20-s trials were recorded for which the average root mean square error (RMS) was used as a measure of deviation between actual and target force. In a force change subtask, two stationary horizontal lines (4 N and 8 N) were displayed on the monitor and the task was to move the vertical bar in between these two target lines by increasing and reducing grip force as fast as possible. Participants completed three eight-second trials, whereby the emphasis of the instruction was on speed rather than accuracy. The average frequency of force change (FCFreq) served as outcome parameter.

Subjects’ performance in the 9-Hole-Peg Test (9HPT) was used as a measure of manual dexterity (Mathiowetz et al., 1985). A Rolyan 9-Hole-Peg Test Kit was centered in front of the participants with the shallow dish on their dominant hand side. Across three trials, the subjects’ task was to place the pegs, one at a time, as quickly as possible into the peg board and subsequently remove them, again one by one, to put them back into the dish. The mean duration across trials was used as outcome measure.

Postural Control was assessed across three different conditions using a Bertec triaxial force plate (Bertec Corp, Columbus, USA) sampling at 600 Hz. Each condition comprised three 30-s trials, in which subjects performed a tandem stance with their dominant leg in front. The three conditions were: (a) eyes closed on a firm surface; (b) eyes open on an Airex^®^ Balance-Pad (Airex AG, Sins, Switzerland); and (c) eyes open on a firm surface while simultaneously performing a visual letter variant of the 2Back Task. While the first two conditions constitute integral parts of the Balance Error Scoring System (BESS), a commonly administered multi-step test to assess postural control following sport-related head impacts (Azad et al., 2016), dual-task protocols (c) have been suggested to serve as particularly sensitive means to detect balance deficits as a result of concussion (Kleiner et al., 2018). Using custom MATLAB routines (MATLAB R2021a, The MathWorks, Natick, USA) center of pressure (CoP) data were processed using a 4th order low-pass Butterworth filter (cut-off: 10 Hz). Across all trials of each condition, the mean displacement velocity of the CoP (CoPV) and the mean distance travelled by the CoP (CoPDist) were calculated to quantify balance performance (Raymakers et al., 2005). In the dual-task condition (c), players stood on the force plate and were presented with letters on a 17″ computer screen, one at a time, and were instructed to respond to the displayed letter if it matched the one shown two trials previously by clicking the left button of a wireless computer mouse. Twenty letters (four targets, 16 distractors) were displayed to match the duration of the balance assessment. Performance parameters for the 2Back Task were the mean reaction time for target stimuli (2Back RT) and the average proportion of correct responses (Accuracy) across trials (Table 2). Due to technical problems, baseline postural control data was corrupted and could not be used for analysis. Therefore, soccer players’ balance performances during the actual re-test served as baseline values.

### Statistical analysis

Statistical analyses were carried out using R (R Core Team, 2022) for Windows. The α-level was set to 0.05 throughout. Effect sizes are indicated in terms of partial eta square ($\eta_{p}^{2}$) or Cohen’s *d*. Unless otherwise stated, descriptive data are presented as means (± SD). To describe our sample’s overall heading behavior, we calculated the average number of headers per player per match. This was done be first dividing the number of headers in each single match by the number of players that actually participated in the respective matches, to then average the obtained values across the 37 matches. To further account for interindividual differences between players in their average number of headers, we also calculated each player’s average number of headers across all of their matches. This was done by dividing each player’s total number of headers by her individual number of matches. To examine the effects of soccer heading on the collected variables of subjective health, concussion symptoms, as well as cognitive and sensorimotor performances, we performed two different, yet in part complementary, analyses.

Given that data of the soccer players and the control subjects were available for the same time interval of one-and-a-half years (baseline—post-test), we (1) compared health perception, concussion symptoms as well as cognitive and sensorimotor performances between groups over time. Therefore, we used the median number of video-identified headers performed by the soccer players that completed both the baseline and post-test measurement session (*n* = 15) to divide the soccer sample into high-exposure and low-exposure subgroups. We used this median-split to account for the interindividual variability in the players’ individual numbers of performed headers and further ensure an equal size of both subgroups. For the time-dependent comparison of both health status (SF-36) and concussion symptoms (SCAT) between the control group, the low-exposure group, and the high-exposure group, we used a cumulative link model (CLM) and two-way mixed analyses of deviance, given the ordinal nature of the response variables. Exposure (control, low-exposure, high-exposure) served as between-subject factor, whereas time (baseline, post-test) was used as within-subject factor. Players’ number of previously sustained head injuries, age at baseline, educational level, and years of soccer experience were used as covariates to account for potential effects. In case of a statistically significant exposure × time interaction, simple main effects were assessed by means of a Kruskall–Wallis test with Bonferroni–Holm-corrected Mann–Whitney tests (differences between groups) and Wilcoxon signed-rank tests (differences within groups across time). Similarly, 3 × 2 mixed ANOVAs were used to compare cognitive and sensorimotor performances between the three subgroups over time, while, again, controlling for potential effects of the number of previously sustained head injuries, age at baseline, educational level, and years of soccer experience. In case of a statistically significant interaction, simple main effects were assessed by means of a one-way ANOVA with Bonferroni–Holm-corrected independent *t*-tests (differences between groups) and dependent *t*-tests (differences within groups across time). In the rare cases of outliers (as defined by the 1.5*IQR rule), we additionally computed the ANOVAs on outlier-free data. However, as the obtained results, essentially, did not differ from each other (i.e., no changes in statistical significance or major changes in the obtained effect sizes), we included the outliers in our analysis in order to not further reduce the amount of data.

To take advantage of the fact that data from more behavioral tests were available for (only) the soccer group between the re- and post-test and since we could include players for whom baseline data were lacking (plus *n* = 7), we (2) used correlation and regression analyses to examine the relationship between soccer heading and changes in cognitive and sensorimotor test performances within this time interval. To that aim, soccer players’ re-test value was subtracted from their corresponding post-test value to obtain a measure of individual performance change (Δ-performance) across the second soccer season. Pearson’s correlation analyses were conducted to statistically analyze an association between the individual number of headers and Δ-performances on the behavioral tests. To further assess the association between player’s individual heading exposure and performance changes across all behavioral tests during the second soccer season (re-test—post-test), a multiple linear regression analysis was conducted. Specifically, we aimed to predict the number of individually performed headers from the players’ Δ-performances across all tests. Within this context, players’ number of previously sustained head injuries, age at baseline, educational level, and years of soccer experience were, again, used as control variables (critical variance inflation factor [VIF] = 5.00) to account for potential effects.

## Results

Intraclass correlation coefficient (ICC) analysis revealed an excellent interrater agreement (Koo and Li, 2016) for the five randomly-selected matches that were reviewed by two researchers (κ = 0.96, 95% CI [0.94–0.97]). Consequently, we considered a single rater to be appropriate for determining heading exposure from video data. Over the entire observational period, soccer players performed a total of 1,425 purposeful headers (first [half] season: 529 headers; second season: 896 headers), which corresponds to an average number of 38.5 (± 11.4) header per match. The total number of headers per player varied between 0 and 263. The average number of headers per player per match was 3.1 (± 2.8), with players’ individual average exposure varying between 0.0 and 7.3 headers per match. No concussive events or unintentional head impacts (e.g., head-to-head, head-to-ground, head-to-goalpost) were observed during any of the soccer matches. Similarly, neither any of the soccer players nor of the control subjects reported suffering a head injury throughout the observational period.

Due to player fluctuations resulting from athletes leaving the team or terminating their soccer career after the end of the first season, 15 soccer players and 12 control subjects completed both the baseline and the post-test assessment. The median number of video-identified headers that these soccer players performed over the entire observational period was 56. Therefore, eight soccer players were allocated to the high exposure group (more than 56 purposeful headers), whereas seven soccer players were assigned to the low exposure group (56 or fewer purposeful headers). Characteristics of the subgroups are displayed in Table 4. There were no statistically significant differences between the three subgroups in any of the demographic, anthropometric, and sport-specific variables, as revealed by one-way ANOVAs.

**TABLE 4:** Subgroups’ characteristics.

	High exposure	Low exposure	Controls
Subjects [n]	8	7	12
Age [y]	21.8 (± 2.9)	22.0 (± 4.34.7)	23.9 (± 3.1)
Height [cm]	166.8 (± 3.9)	169.9 (± 4.3)	169.2 (± 5.9)
Mass [kg]	60.0 (± 5.7)	63.3 (± 3.5)	64.2 (± 6.4)
Years of training [y]	16.3 (± 2.6)	16.1 (± 6.0)	13.4 (± 4.8)
Training/week [h]	4.9 (± 0.9)	5.9 (± 1.9)	4.7 (± 1.4)
Head injuries [n]	0.9 (± 1.0)	0.3 (± 0.5)	0.4 (0.5)
Headers [n]	111.1 (± 51.4)	23.1 (± 19.9)	–

Values depicted as mean (± SD).

The subgroups’ median scores of self-perceived health (SF-36) and concussion symptom scores (SCAT3) in the baseline and post-test assessments are depicted in Table 5. With respect to participants’ subjective health status, analyses of deviance showed no statistically significant exposure × time interactions for any of the eight SF-36 subscales. There only was a tendency toward an interaction of the two factors for the social functioning subscale (*p* = 0.057), with the high exposure subgroup showing a deterioration in self-perceived levels of social functioning, whereas the control and low exposure groups’ scores increased from baseline to post-test. While analyses also showed no statistically significant main effects of heading exposure or time on subjects’ self-perceived health, analysis of the covariates revealed statistically significant effects of the number of previously sustained head injuries on the dimensions of physical functioning (*p* = 0.022), pain (*p* = 0.007), and general health (*p* = 0.006). In all cases, a greater number of head injuries was associated with lower scores (i.e., lower levels of health) on the respective subscales. Similarly, analysis of concussion symptoms, as assessed using the SCAT3, showed no statistically significant interaction effect of the two factors exposure and time on the total symptom score. However, consideration of the covariates revealed a trend pointing toward an effect of the number of head injuries on concussion symptoms (*p* = 0.055), with a greater number of previous head injuries being associated with a higher (i.e., worse) symptom score.

**TABLE 5:** Concussion symptoms (SCAT3) and self-perceived health status (SF-36) of the three subgroups in the baseline and post-test assessments.

	High exposure		Low exposure		Control	
	**Baseline**	**Post-test**	**Baseline**	**Post-test**	**Baseline**	**Post-test**
**Concussion symptoms (SCAT3)**						
Total symptom score	6.0 ± 4.3	8.0 ± 12.0	7.0 ± 19.0	6.0 ± 4.5	6.5 ± 7.0	10.5 ± 13.8
**Subjective health (SF-36)**						
Physical functioning	100 ± 1.3	100 ± 0.0	100 ± 2.5	100 ± 0.0	100 ± 3.8	100 ± 0.0
Role limitations physical	100 ± 6.3	100 ± 6.3	100 ± 0.0	100 ± 0.0	100 ± 0.0	100 ± 0.0
Role limitations emotional	100 ± 0.0	100 ± 0.0	100 ± 0.0	100 ± 0.0	100 ± 33.0	100 ± 8.3
Energy/fatigue	67.5 ± 18.8	67.5 ± 6.3	70.0 ± 25.0	65.0 ± 10.0	62.5 ± 20.0	67.5 ± 21.3
Emotional wellbeing	79.0 ± 25.0	84.0 ± 26.0	80.0 ± 12.0	80.0 ± 8.0	74.0 ± 20.0	76.0 ± 14.0
Social functioning	100 ± 18.3	87.5 ± 40.3	100 ± 31.0	100 ± 0.0	100 ± 31.3	94.0 ± 25.0
Pain	100 ± 0.0	100 ± 2.5	100 ± 0.0	100 ± 16.0	85.0 ± 24.5	84.0 ± 32.0
General health	85.0 ± 23.8	92.5 ± 13.8	80.0 ± 12.5	80.0 ± 7.5	75.0 ± 31.3	85.0 ± 26.3

Values depicted as median (± IQR). Outcomes were controlled for the number of previous head injuries, educational level, player age at baseline, and years of soccer experience. SCAT3: higher values indicate greater symptom burden (maximum score: 132). SF-36: higher values indicate more favorable level of self-perceived health (maximum score: 100).

**FIGURE 1:**
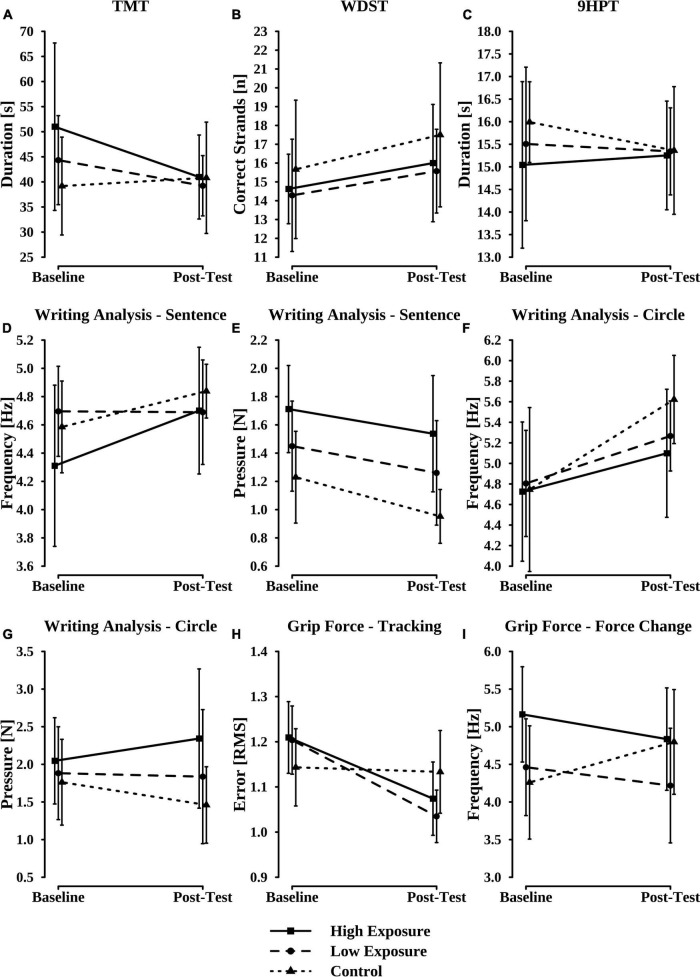
Average baseline and post-test performances achieved by the high exposure group (solid lines), the low exposure group (dashed lines), and the control group (dotted lines) in the Trail Making Test **(A)**, the Wechsler Digit Span Test **(B)**, the 9-Hole-Peg Test **(C)**, the sentence subtask of the handwriting analysis **(D,E)**, the circle subtask of the handwriting analysis **(F,G)**, the tracking subtask of the grip force analysis **(H)**, and the force change subtask of the grip force analysis **(I)**.

The mean performance scores that were achieved by the three subgroups across the cognitive and sensorimotor tests over time are displayed in Figure 1 and Table 6. For the TMT, the ANOVA revealed a trend toward an exposure × time interaction (*p* = 0.051, $\eta_{p}^{2}$ = 0.24). While both subgroups of soccer players, on average, performed worse (i.e., longer duration) than the control group during baseline assessments, all groups showed similar performances during the post-test (Figure 1A). Analysis of the subgroups’ performances in the WDST showed a statistically significant time effect, *F*_1,24_ = 6.03, *p* = 0.022, $\eta_{p}^{2}$ = 0.20, as indicated by an increase in the average number of strands correctly reproduced by each group (Figure 1B). Concerning the groups’ performances in the 9HPT, the conducted ANOVA neither revealed a statistically significant interaction effect, nor main effects of the two factors exposure and time (Figure 1C). With respect to writing frequency (SFreq) during the sentence subtask of the analysis of handwriting, there was neither a statistically significant interaction between exposure and time nor a significant main effect of the two factors (Figure 1D). For the exerted pen pressure (SPress), however, we found statistically significant main effects of both exposure, *F*_2,24_ = 10.48, *p* < 0.001, $\eta_{p}^{2}$ = 0.47, and time, *F*_1,24_ = 10.80, *p* = 0.003, $\eta_{p}^{2}$ = 0.31, but no significant interaction effect (Figure 1E). While the time effect indicated a significant performance increase (i.e., decrease in pen pressure) from baseline to post-assessment across all subgroups, post-hoc independent *t*-tests with Bonferroni–Holm adjustments revealed that average pen pressure of the control group was significantly lower compared to the high exposure group (*p* = 0.001, *d* = 1.82) and also lower than the low exposure group’s average values (*p* = 0.064, *d* = 0.95). Moreover, a *t*-test showed a tendency toward an increased pen pressure in the high exposure group as compared to the low exposure group (*p* = 0.068, *d* = 0.87). For the circle subtask of the writing analysis, the conducted ANOVA showed a statistically significant main effect of time on the frequency parameter (CFreq), *F*_1,24_ = 16.54, *p* < 0.001, $\eta_{p}^{2}$ = 0.41, with average frequency increasing (i.e., more favorable performance) from baseline to post-test across all groups (Figure 1F). With respect to pen pressure during the circle subtask (CPress), we found a trend toward an exposure × time interaction (*p* = 0.068, $\eta_{p}^{2}$ = 0.20). While all groups showed comparable performances at baseline, average pen pressure tended to decrease (i.e., rather favorable development) in the control group, remained similar in the low exposure group, and increased in the high exposure subgroup (Figure 1G). Analysis of the performances achieved by the different subgroups during the first grip force task (Tracking) revealed a statistically significant interaction effect of exposure and time on the RMS as dependent variable, *F*_2,24_ = 10.41, *p* < 0.001, $\eta_{p}^{2}$ = 0.47. Dependent *t*-tests showed a statistically significant simple main effect of time on the average performance of the low exposure (*p* = 0.010, *d* = 1.40) as well as the high exposure subgroup (*p* = 0.002, *d* = 1.68), with both groups displaying decreases in the RMS (i.e., performance increase) from baseline to post-test. In contrast, the control group’s average performance remained rather stable over time (Figure 1H). For the grip force change subtask, there was a tendency toward an exposure × time interaction on the dependent variable FCFreq (*p* = 0.056, $\eta_{p}^{2}$ = 0.21). While the control group showed an increase in force change frequency over time (i.e., rather favorable development), both exposure groups displayed decreases in FCFreq from baseline to post-test (Figure 1I). None of the considered covariates (number of previous head injuries, age at baseline, educational level, years of soccer experience) was found to have a statistically significant effect on the behavioral outcomes.

**TABLE 6:** Test performances achieved by the three subgroups in the baseline and post-test assessments.

Variables	High exposure (*n* = 8)		Low exposure (*n* = 7)		Control (*n* = 12)		ANOVA effects		
	**Baseline**	**Post-test**	**Baseline**	**Post-test**	**Baseline**	**Post-test**	**Interaction**	**Exposure**	**Time**
TMT [s]	51.0 ± 16.9	40.9 ± 9.0	44.3 ± 8.9	40.3 ± 5.9	38.8 ± 10.9	40.8 ± 11.1	*p* = 0.051	*p* = 0.442	*p* = 0.060
WDST [n]	14.6 ± 1.8	16.0 ± 3.1^a^	14.2 ± 3.0	15.6 ± 2.2^a^	15.9 ± 4.1	17.5 ± 3.8^a^	*p* = 0.974	*p* = 0.390	*p* = 0.022*, $\eta_{p}^{2}$ = 0.20
9HPT [s]	15.0 ± 1.8	15.3 ± 1.2	15.5 ± 1.7	15.3 ± 1.0	15.9 ± 1.0	15.4 ± 1.4	*p* = 0.429	*p* = 0.696	*p* = 0.514
SFreq [Hz]	4.3 ± 0.6	4.7 ± 0.4	4.7 ± 0.7	4.7 ± 0.8	4.7 ± 0.5	4.8 ± 0.4	*p* = 0.240	*p* = 0.510	*p* = 0.088
SPress [N]	1.7 ± 0.3^b^	1.5 ± 0.4^a^^b^	1.5 ± 0.3	1.3 ± 0.4^a^	1.2 ± 0.3^b^	1.0 ± 0.2^a^^b^	*p* = 0.957	*p* < 0.001*, $\eta_{p}^{2}$ = 0.47	*p* < 0.001*, $\eta_{p}^{2}$ = 0.31
CFreq [Hz]	4.7 ± 0.7	5.1 ± 0.6^a^	4.8 ± 0.5	5.3 ± 0.3^a^	4.9 ± 0.8	5.6 ± 0.4^a^	*p* = 0.525	*p* = 0.275	*p* < 0.001*, $\eta_{p}^{2}$ = 0.41
CPress [N]	2.0 ± 0.6	2.3 ± 0.9	1.9 ± 0.6	1.8 ± 0.9	1.7 ± 0.5	1.5 ± 0.5	*p* = 0.068	*p* = 0.122	*p* = 1.000
GFTrack [RMS]	1.2 ± 0.1	1.1 ± 0.1^a^	1.2 ± 0.1	1.1 ± 0.1^a^	1.1 ± 0.1	1.1 ± 0.1	*p* < 0.001*, $\eta_{p}^{2}$ = 0.46	*p* = 0.792	*p* < 0.001*, $\eta_{p}^{2}$ = 0.54
FCFreq [Hz]	5.2 ± 0.6	4.8 ± 0.7	4.5 ± 0.6	4.2 ± 0.8	4.4 ± 0.8	4.8 ± 0.7	*p* = 0.056	*p* = 0.127	*p* = 0.745

^a^Significant time effect between baseline and post-test.

^b^Significant difference between the high exposure and control group at the given timepoint.

*p < 0.05. Values depicted as mean ± SD. Outcomes were controlled for the number of previous head injuries, educational level, player age at baseline, and years of soccer experience. TMT, Trail Making Test; WDST, Wechsler Digit Span Test; 9HPT, 9-Hole-Peg Test; S, sentence; Freq, frequency; C, circle; Press, pressure; GF, grip force; Track, tracking; FC, force change.

**FIGURE 2:**
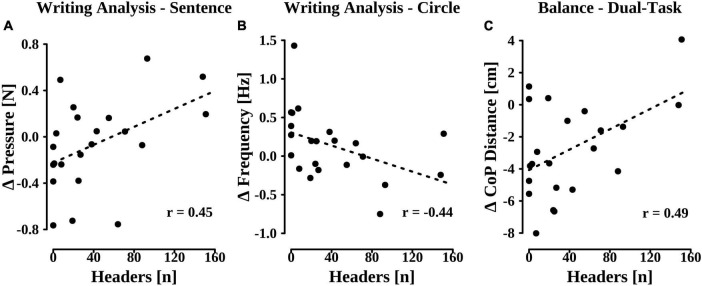
Changes in pen pressure during the sentence subtask **(A)**, frequency during the circle subtask **(B)**, and distance covered by the center of pressure (CoP) during the dual balance task **(C)** across the second soccer season in relation to players’ individual number of performed headers.

Pearson’s correlation analyses were used to statistically analyze a potential association between soccer players’ (*n* = 22) individual number of performed headers and changes in their cognitive and sensorimotor test performances across the second season (re-test—post-test). For the majority of parameters, there were no statistically significant associations. However, we found statistically significant linear relationships between players’ individual heading exposure and negative changes in both writing subtasks as well as in the dual balance task (Figure 2). Specifically, a greater number of headers was associated with an increased pen pressure in the sentence subtask of the writing analysis (*r* = 0.45, *p* = 0.034; Figure 2A), a frequency decrease in the circle subtask (*r* = −0.44, *p* = 0.043; Figure 2B), and an increase in the distance covered by the CoP during the dual balance task (*r* = 0.49, *p* = 0.021; Figure 2C).

Ultimately, a multiple linear regression analysis was conducted to investigate a potential link between heading exposure and performance changes across all behavioral tests. More specifically, it was assessed whether the individual numbers of performed soccer headers could be predicted by the observed cognitive and sensorimotor performance changes between re- and post-test while controlling for the effects of previously sustained head injuries, age at baseline, and years of soccer experience. Analysis revealed a statistically significant regression model (*R*^2^_adj_ = 0.65, *p* < 0.001) with four factors (Table 7). In line with the results of the correlation analyses, we found that greater numbers of individually performed headers came along with an increase in pen pressure during the sentence subtask (*ß* = 0.35, *p* = 0.022), a decrease in writing frequency during the circle subtask (*ß* = −0.49, *p* = 0.002), and an increase in CoP distance in the balance subtask that was performed in conjunction with the 2Back-Task (*ß* = 0.49, *p* = 0.002). However, regression results further showed that, next to the unfavorable development of balance performance, greater heading exposure was also associated with an improved 2Back-Task performance, as indicated by an accuracy increase (*ß* = 0.35, *p* = 0.023). None of the covariates contributed significantly to the model.

**TABLE 7:** Multiple linear regression model (*R*^2^_adj_ = 0.65, *p* < 0.001) for the prediction of soccer players’ individual number of headers from the observed cognitive and sensorimotor performance changes between re-test and post-test.

Parameter	ß	*p*
Δ Writing analysis SPress [N]	0.347	0.022*
Δ Writing analysis CFreq [Hz]	−0.488	0.002*
Δ Balance DT CoPDist [cm]	0.486	0.002*
Δ 2Back-Task accuracy [%]	0.351	0.023*

Outcomes were controlled for the number of previous head injuries, educational level, player age at baseline, and years of soccer experience.

*p < 0.05.

Given the overall small sample size and its further reduction due to player fluctuation and drop-outs, we conducted post-hoc power analyses using G*Power 3.1 (Heinrich-Heine University Düsseldorf, Germany) (Faul et al., 2009) to gain a better understanding of our analyses’ statistical power. For the non-significant subgroup (ANOVA) analyses, statistical power ranged from 0.06 to 0.69. For the statistically significant subgroup differences in pen pressure during the handwriting analysis, results revealed a substantial statistical power of 0.99. Likewise, a considerable power of 0.99 was obtained for the results of the multiple regression analysis.

## Discussion

Within recent years, the proliferate concern about the consequences of sport-related head impacts has led to an increasing amount of research on soccer heading and its potentially adverse effects on brain function. The aim of this paper was to expand this research by focusing on the largely underrepresented group of higher-level female athletes. In a prospective approach, self-perceived health, concussion symptoms, and, above all, cognitive and sensorimotor skills of semi-professional female soccer players and control athletes participating in non-contact sports were assessed at baseline and after one-and-a-half soccer seasons, during which soccer players’ heading exposure was determined by analyzing video footage of competitive matches. Overall, comparisons between a high exposure, low exposure, and control group over time as well as correlation analyses between soccer players’ individual number of headers and their exhibited changes in cognitive and sensorimotor test performances yielded no unequivocal evidence for negative consequences of soccer heading. Still, we found some indications pointing toward subtle adverse effects of heading on aspects of fine motor and postural control that warrant further attention.

Across the 37 soccer matches that were analyzed for the purpose of this study, we observed an average number of 39 headers per match, which corresponds to an average exposure of about three headers per player per match. Compared to the exposure rates of one only header per player per match that have been reported in female soccer players at youth (Bonn et al., 2021) or high school level (Kaminski et al., 2007, 2008), these numbers present a noticeable increase in heading frequency and, thus, confirm the notion that cumulative exposure to RHI due to purposeful heading might be greater in female soccer athletes per-forming at higher levels of play (Tarnutzer et al., 2017). In turn, average heading exposure of our female sample was lower than that reported by Cassoudesalle et al. (2020) for their group of semi-professional male players (49.2 headers per match, 3.4 headers per player per hour). While this is in line with the notion that male soccer players perform more headers than females (Langdon et al., 2022), we observed a considerable interindividual variability in the number of headers, with single players, on average, experiencing more than seven headers per match—rates that are quite comparable to those reported for male athletes at semi-professional level (Cassoudesalle et al., 2020). In conjunction with the increased vulnerability of women toward RHI (Bretzin et al., 2017; Caccese et al., 2018; Rubin et al., 2018), this confirms our rationale for explicitly focusing on higher level female athletes in our investigation of the potentially adverse effects of soccer heading.

On a subjective level, we found no time-dependent differences in health status (SF-36) and concussion symptoms (SCAT3) between frequent and rare headers as well as subjects with no exposure to soccer heading. At first, this can be seen as a contradiction to the findings of Prien et al. (2020), who observed worse self-perceived health outcomes with increasing heading exposure. However, it must be noted that their study, in contrast to our approach, focused on a group of retired athletes. Thus, it could be argued that repetitive heading does not lead to an immediate or short-term deterioration of subjective health levels in young and active individuals, but that the (potentially) subtle effects of RHI from heading the ball might accumulate over the course of a playing career and result in a reduction of self-perceived health status in older age. However, since other factors associated with prolonged (contact) sports participation, such as musculoskeletal injuries or osteoarthritis, have also been suggested to negatively affect health perception of retired athletes (Filbay et al., 2019), the relationship between RHI from soccer heading and a long-term reduction of self-perceived health status needs further investigation. A worse perception of health on the SF-36 subscales of physical functioning, pain and general health as well as SCAT3 concussion symptoms was, however, associated with greater numbers of previously sustained head injuries—an observation, which is consistent with previous findings indicating that healthy athletes with a history of sport-related head injuries display higher concussion symptom scores (Snedden et al., 2017) and an inferior perception of health-related domains, such as physical functioning, bodily pain, or health in general (Valovich McLeod et al., 2010) as compared to non-concussed subjects. Within this context, statistically significant negative correlations between subjects’ SCAT3 total symptom score and the corresponding SF-36 scores in the domains of physical functioning (*r*_s_ = −0.38, *p* = 0.005), pain (*r*_s_ = −0.40, *p* = 0.003), and general health (*r*_s_ = −0.34, *p* = 0.011) suggest that the persistent prevalence of subtle concussion-related symptoms could be reflected in lower levels of subjective health.

With respect to the main aim of our study, the prospective examination of the effects of headers on selected cognitive and sensorimotor domains, two steps of analysis were performed. On the one hand, behavioral test performances were compared between soccer players with low and high heading exposure and a control group of subjects from non-contact sports before and after a period of one-and-a-half years. On the other hand, we used correlation and multiple linear regression analyses to assess the relationship between female soccer players’ number of individually performed headers and Δ-test performances from an extended test battery across the second season. While the results of several previous approaches hinted toward a dose-response-relationship between cumulative soccer heading over a single season and the degree of adverse functional brain changes (Matser et al., 2001; Lipton et al., 2013), neither of our statistical approaches revealed unequivocal evidence for negative consequences of purposeful heading on the here assessed cognitive and sensorimotor domains. Concerning the results of our subgroup analyses, some measures, such as manual dexterity (9HPT), appeared to be entirely unaffected by both heading exposure and time, whereas performances in other domains—e.g., memory (WDST)—even increased across all groups. In their prospective approaches, both Kaminski et al. (2008) and Wahlquist et al. (2019) observed similar improvements in female soccer players’ cognitive performances (including memory) over time and attributed these developments to practice effects resulting from multiple testing sessions—an interpretation that might be equally valid for our data. Within this context, it has to be also considered that soccer players took part in three measurement sessions (baseline, re-test, post-test), whereas control subjects could be assessed only twice (baseline, post-test). Therefore, greater learning effects may have been present for the soccer athletes as compared to the control subjects. This might be seen as an explanation for the unexpected finding of a statistically significant group × time interaction in the grip force tracking subtask. While both soccer subgroups showed performance increases (reduced RMS) from baseline to post-test, the control group’s performance remained rather stable over time. For this exact task, previous research could show significant learning effects over multiple testing sessions (Kriz et al., 1995). The same explanation might be equally valid for the borderline significant group × time interaction in the TMT—a task in which performance has been also reported to be positively affected by practice (Buck et al., 2008). However, even when accounting for these practice effects by correlating soccer players’ number of individually performed headers with Δ-test performances across the second season (extended test battery), there appeared to be no conclusive evidence for a relationship between heading exposure and negative developments of cognitive and sensorimotor performances. Still, when interpreting these findings, the relatively young age of our soccer sample should be taken into account. Until the mid-twenties, there are ongoing re-organization and myelinization processes within the central nervous system (Bove, 2018) that might act as neural repair mechanisms potentially counteracting the presumed adverse effects of RHI from soccer heading. In other words, it might be the case that repetitive headers do have a negative effect on soccer players’ cognitive or sensorimotor function, but these effects do not become apparent in younger individuals, as they are compensated by these neural repair mechanisms.

Since previous studies predominantly focused on the evaluation of cognitive alterations as a potential result of soccer-related RHI (Lipton et al., 2013; Prien et al., 2020; Ashton et al., 2021), it is noteworthy that the few indications pointing toward subtle adverse effects of heading in our data could rather be attributed to the sensorimotor domains of fine motor and postural control. Such potential long-term or cumulative consequences of RHI due to heading on motor control are largely unexplored. With respect to the domain of fine motor control, we found that soccer players exhibited a consistently greater pen pressure than the control group during the sentence subtask of the writing analysis, while the high exposure group also displayed increases in pen pressure over time during the subsequent circle subtask. To the authors’ knowledge, these tasks have not been used in the direct context of head injury or head impact research. Increases in pen pressure during handwriting have, however, been reported in patients with writer’s cramp (Hermsdörfer et al., 2011) and interpreted as a symptom indicative of focal dystonia (Pirio Richardson et al., 2017) as well as in individuals suffering from parkinsonism (Saini and Kaur, 2019). Like Parkinson’s disease, focal dystonia has been linked to a dysfunction of the basal ganglia (Pirio Richardson et al., 2017). In turn, parkinsonism has been commonly identified as a (late) consequence of RHI, especially in boxers (Davie et al., 1995). While this hypothetical explanation might illustrate a potential link between our header-related findings and previous reports about the presumed mechanisms underlying motor impairments in patients or former contact sport athletes, the present results do not provide evidence for such a dysfunction of the basal ganglia and, thus, should not be over-interpreted as such. This is especially true as we did not find associations between the number of previous head injuries and fine motor performance changes in our sample. Moreover, given that the observed changes in pen pressure were rather subtle and the soccer players’ fine motor performances do not represent clinically relevant deteriorations, this interpretation should be taken with caution—especially when considering our small sample size. Interestingly, however, these indications of adverse effects of soccer heading on fine motor control during handwriting were confirmed by the correlation and multiple regression analyses. Considering the latter, our regression model was able to explain a considerable 65 % of the substantial variance within the number of individually performed headers across the second season (range: 0–151). Within this context, three out of four factors could be attributed to sensorimotor domains, which further supports the notion of potential alterations in motor control due to RHI. Next to unfavorable changes in fine motor control during the writing tasks, the regression model revealed an association between players’ individual heading exposure and negative alterations in postural control in the dual-task condition. In contrast to the assessment of fine motor skills, postural control tasks have been more commonly used to evaluate the potentially detrimental effects of soccer heading. However, deteriorations in postural control have been identified in the acute phase following a controlled bout of soccer heading rather than between pre- and post-season assessments, as concluded in a recent systematic review (Bonke et al., 2020). While our results, thus, present the first to point toward a subtle cumulative effect of repetitive soccer heading on postural control over an extended period of time, it has to be noted that the majority of players showed improvements in balance performance (reduced CoP distance) and that a greater number of individually performed headers was rather associated with reduced performance increases. Therefore, results should not be interpreted to present the onset of a progressive deterioration of postural control due to repetitive heading—especially since the observed changes in balance performance came along with an increased working memory performance (2Back-Task Accuracy). Nevertheless, the here adopted dual-task paradigm might provide a sensitive means for uncovering the potentially adverse effects of RHI on measures of postural control, as also previously suggested by Kleiner et al. (2018) in the context of concussion assessment. In summary, while the few indications pointing toward subtle effects of heading on fine motor and postural control should not be over-interpreted to represent clinically meaningful deteriorations in sensorimotor integration, they warrant further attention in a sense that future large-scale studies should not only focus on cognitive outcomes, but also consider sensorimotor changes as a potential consequence of RHI from heading the ball.

### Limitations

Although our study aimed for a careful implementation of methodological recommendations from previous research (Rodrigues et al., 2016; Tarnutzer et al., 2017), such as the employment of a prospective design, a high-quality assessment of heading exposure, the consideration of the effects of previously sustained head injuries, or the inclusion of control subjects with similar physical activity profile as compared to the group of soccer players, it has several limitations that need to be taken into account when interpreting the results. First and foremost, statistical power, and thus generalizability of our findings, may have suffered from the rather low sample size—especially with respect to our subgroup analyses. Within this context, we also faced the problem of player fluctuations, i.e., players leaving or joining the team between behavioral measurements, which contributed to the reduction of our initial sample size. To gain a better understanding of our analyses’ statistical power, we conducted post-hoc power analyses for the ANOVA and multiple linear regression approaches using the obtained effect sizes and determination coefficients, respectively. On the one hand, results revealed that, for the non-significant subgroup (ANOVA) analyses, statistical power was rather low (range: 0.06–0.69), indicating the need for greater sample sizes. On the other hand, for the significant subgroup differences in pen pressure during the analysis of handwriting, we observed a considerable statistical power of 0.99. Similarly, a high power of 0.99 was obtained for the multiple regression results, suggesting that the observed results might be present. While the recruitment of a greater number of soccer players from different teams could have compensated for player fluctuation and drop-out, it would have made it difficult to ensure a reliable quantification of heading exposure due to the considerable personal, logistical, and financial effort associated with the self-organized recording and analysis of video footage. Therefore, we opted for choosing a high-quality assessment of heading exposure over a greater sample size. A second limitation lies in the exclusive focus on competitive matches for the determination of players’ heading frequency. It could be argued that, due to heading in training sessions, individual numbers of performed headers were actually higher than reported. While this can be considered a valid point, we were assured that the team’s training sessions did not encompass any specific heading drills. Moreover, by assuming a constant intraindividual heading behavior in small-sided games during practices, we are confident that the neglect of headers performed during training sessions neither affected the composition of exposure groups nor the interindividual exposure ratio with respect to the performed correlation analyses. Another issue that should be taken into account is the rather short observational period of one-and-a-half years. By assuming rather subtle cumulative effects of RHI from heading the ball, it is conceivable that any adverse outcomes might only become apparent after more extended periods. However, the duration of our study, still, exceeded the ones of other prospective approaches (Kaminski et al., 2007; Bonn et al., 2021) and, thus, our project can be considered a valuable addition to the existing body of research on the potential effects of repetitive soccer heading.

## Conclusion

Although we observed greater numbers of intentional headers in our sample of semi-professional female soccer players as compared to women competing at youth or high school level, we did not find conclusive evidence for negative consequences of purposeful headers on players’ perception of health, concussion symptoms as well as cognitive and sensorimotor performances over a period of one-and-a-half years. However, there were some indications for subtle negative effects of repetitive heading on aspects of fine motor control during a handwriting task and postural control—specifically when the latter was assessed using a dual-task paradigm. Given the overall small sample size and its further reduction due to player fluctuation throughout the study period, these findings should not be over-interpreted—especially since the observed performance changes appeared to be rather subtle and, in this sense, do not represent clinically relevant deteriorations in players’ sensorimotor function due to heading. Nevertheless, our results warrant further attention, as previous studies mainly focused on cognitive, but not sensorimotor alterations as a potential result of repetitive soccer headers. In order to gain a deeper understanding of the relationship between RHI and changes in soccer athletes’ brain health, future research should not solely focus on the assessment of cognitive variables, but also consider sensorimotor changes as a potential consequence of heading the ball. Within this context, longer-term prospective studies involving larger samples of (high-level) soccer athletes and a high-quality assessment of players’ heading exposure are imperative to elucidate the potentially adverse cumulative effects of purposeful soccer heading. Besides a focus on behavioral outcomes, these studies might consider multimodal approaches, including MRI scans to assess potential changes in brain structure as well as blood or saliva sampling in order to gain insights into the molecular mechanisms associated with repetitive headers. By this, different stakeholders, such as players, coaching staff, and decision makers, can be provided with important information on the true risk of repetitive soccer headers.

## Data availability statement

The raw data supporting the conclusions of this article will be made available by the authors, without undue reservation.

## Ethics statement

The studies involving humans were approved by the Ethikkommission der Fakultät für Medizin der TU München Ismaninger Str. 22 81675 München. The studies were conducted in accordance with the local legislation and institutional requirements. The participants provided their written informed consent to participate in this study.

## Author contributions

JK: Conceptualization, Data curation, Formal analysis, Investigation, Methodology, Software, Visualization, Writing – original draft. PG: Data curation, Formal analysis, Validation, Writing – review & editing. JH: Conceptualization, Funding acquisition, Project administration, Resources, Supervision, Validation, Writing – review & editing.

## Footnotes

1
https://www.kinovea.org

2
https://www.neurobs.com

## Supplementary material

Supplementary Figure 1 Process of data collection, along with the number of subjects (including drop-outs and drop-ins) that took part in the assessments at the single time-points.
