# Supplementary figures and images for: A prospective investigation of the effects of soccer heading on cognitive and sensorimotor performances in semi-professional female players
Source: Front Hum Neurosci. 2024 Feb 9;18:1345868. doi: 10.3389/fnhum.2024.1345868 (PMC10884128; doi:10.3389/fnhum.2024.1345868)

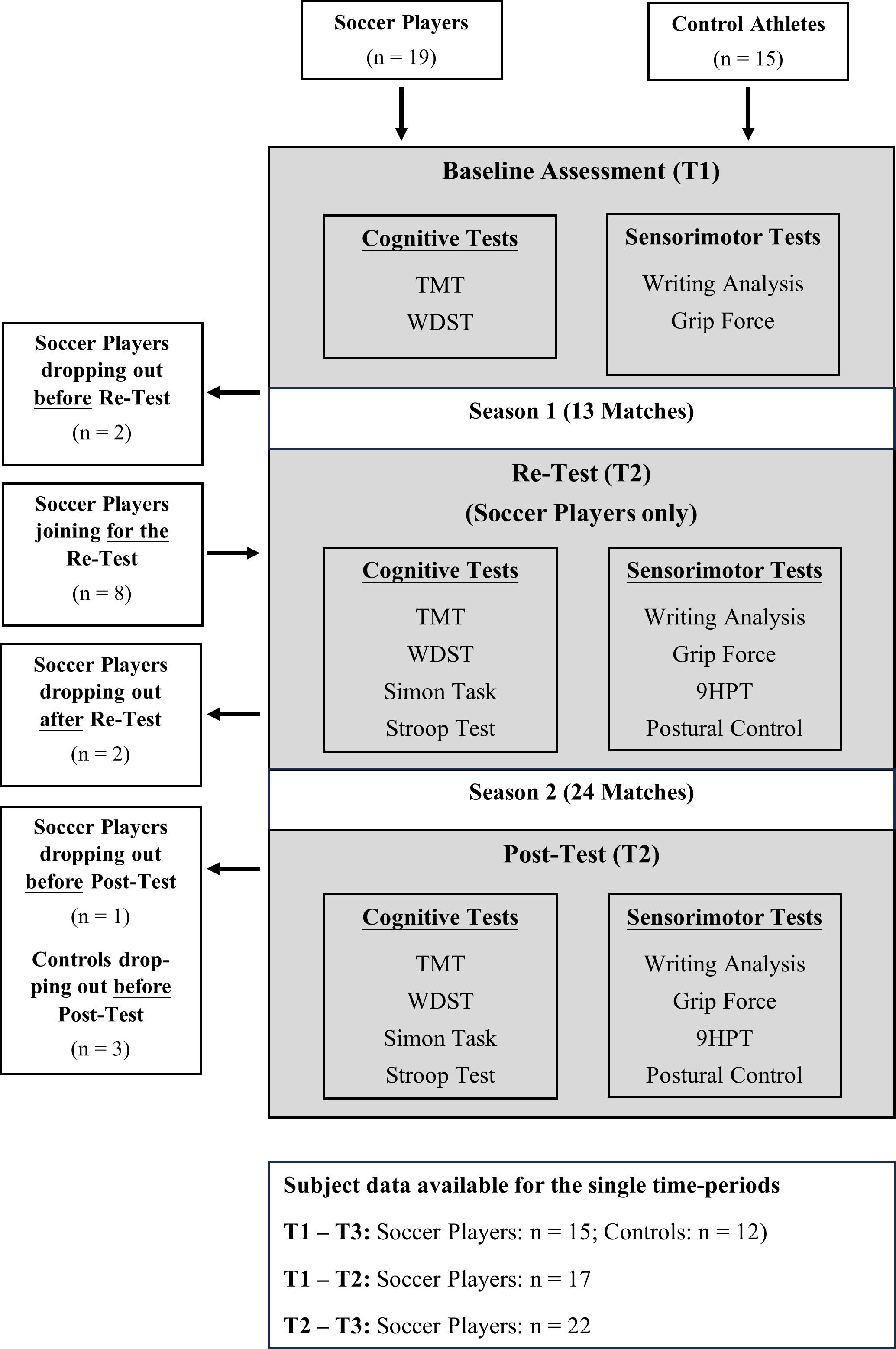

Supplement: Supplementary Figure 1 — Process of data collection, along with the number of subjects (including drop-outs and drop-ins) that took part in the assessments at the single time-points. [file Image_1.jpg]
